# Exosomes Derived From CTF1-Modified Bone Marrow Stem Cells Promote Endometrial Regeneration and Restore Fertility

**DOI:** 10.3389/fbioe.2022.868734

**Published:** 2022-04-13

**Authors:** Qianqian Zhu, Shengluan Tang, Yanwen Zhu, Di Chen, Jialyu Huang, Jiaying Lin

**Affiliations:** ^1^ Department of Assisted Reproduction, Shanghai Ninth People’s Hospital, Shanghai Jiao Tong University School of Medicine, Shanghai, China; ^2^ Center for Reproductive Medicine, Jiangxi Maternal and Child Health Hospital, Nanchang University School of Medicine, Nanchang, China

**Keywords:** cardiotrophin-1, exosomes, bone marrow-derived stem cells, angiogenesis, endometrial regeneration, fertility restoration

## Abstract

**Background:** Thin endometrial tissue is a leading cause of embryo transfer failure, potentially contributing to sustained infertility and associated adverse outcomes. The application of exosomes derived from autologous or allogeneic bone marrow-derived stem cells (BMSCs) has been used to promote uterine repair following injury, and there is also prior evidence that stem cell transplantation can bolster fertility. Genetic modifications represent a primary approach to enhancing exosomal therapy strategies. The present study thus explored the effects of Cardiotrophin-1 (CTF1)-modified BMSCs-exo on fertility-related outcomes.

**Methods:** An adenoviral vector was used to generate CTF1-overexpressing BMSCs (C-BMSCs), after which exosomes were isolated from control BMSCs (BMSC-exos) and C-BMSCs (C-BMSC-exos). The angiogenic effects of C-BMSC-exo treatment were assessed through analyses of endothelial cell proliferation and tube formation. Model rats exhibiting endometrial thinning were administered C-BMSCs-exo, after which the effects of such treatment were assessed through H&E staining, Masson’s trichrome staining, and immunofluorescence analyses. The mechanistic basis for the proangiogenic effects of CTF1 as a driver of endometrial regeneration was additionally explored.

**Results:** C-BMSC-exo treatment of HUVECs was associated with enhanced neovascularization, as evidenced by improved *in vitro* proliferation, migration, and tube formation. Importantly, such treatment was also linked to tissue regeneration, neovascularization, and the suppression of localized tissue fibrosis *in vivo*. Regenerated endometrial tissue exhibited higher embryo receptivity and was associated with higher birth rates in treated rats. The upregulation of the JAK/PI3K/mTOR/STAT3 signaling pathways in C-BMSC-exo-treated rats may underscore the mechanistic basis whereby CTF1 can positively impact endometrial angiogenesis and regeneration.

**Conclusion:** Our data suggest that exosomes produced by CTF1-modified BMSCs can more effectively promote the regeneration of endometrial and myometrial tissues, driving neovascularization in a manner that improves endometrial receptivity in a rat model system, highlighting the therapeutic promise of this approach for patients diagnosed with endometrial thinning.

## Background

Embryo implantation is dependent upon both endometrial receptivity and appropriate embryonic development (Taylor and Gomel, 2008). Endometrial receptivity can be compromised in patients with Asherman syndrome or in individuals with a thin endometrium, resulting in lower rates of implantation and higher rates of spontaneous pregnancy loss (Richter et al., 2007; Lebovitz and Orvieto, 2014). In these patients, low rates of implantation may be linked to impaired radial artery blood flow and the consequent disruption of endometrial glandular growth as a consequence of reduced vascular endothelial growth factor (VEGF) production and associated decreases in angiogenesis (Mahajan and Sharma, 2016). While several pharmacological agents have been used to promote endometrial regeneration in humans, their efficacy is often limited (Lin et al., 2018). The intrauterine administration of granulocyte colony-stimulating factor (G-CSF) is generally the most popular treatment for affected patients, yet its efficacy varies across studies (Gleicher et al., 2013; Barad et al., 2014; Check et al., 2014; Kunicki et al., 2014). Improvements in endometrial vascularization have also been reported in women treated with sildenafil, tocopherol, aspirin, and L-arginine, while prolonged estrogen therapy has been explored as an approach to improving endometrial vascularity. Gonadotropin-releasing hormone (GnRH) analog utilization prior to hormone replacement therapy (HRT) is not recommended, as it can result in reductions in endometrial growth and vascularization (Chen et al., 2013; Mahajan and Sharma, 2016). There is thus an urgent need for the development of more reliable treatments capable of promoting endometrial and myometrial vascularization and regeneration in patients diagnosed with a thin endometrium. Tissue engineering strategies may hold promise as a means of restoring uterine function in these individuals.

Researchers have recently begun to explore the value of uterine cell therapy strategies as a treatment for endometrial dysfunction (Gargett and Ye, 2012). Bone marrow mesenchymal stem cells (BMSCs) are readily isolated multipotent cells capable of differentiating into an array of different cell types (Oswald et al., 2004). Owing to their ability to readily expand *in vitro* and to their immunomodulatory properties (Jing et al., 2014), BMSCs are often studied in the context of tissue regeneration. While there are few BMSCs found in systemic circulation at any given time, they can readily home to the endometrium or other damaged tissue sites (Gargett and Ye, 2012). Human and murine study results have indicated that the incorporation of low numbers of BMSCs into endometrial tissues can promote angiogenesis, and that these cells can additionally undergo transdifferentiation to yield endometrial epithelial, stromal, and endothelial cells (Du and Taylor, 2007; Ikoma et al., 2009; Du and Taylor, 2010).

Despite the promise of stem cell-based therapy, these strategies are limited by the potential for tumorigenicity, emboli formation, immunogenicity, and infectious disease transmission associated with such treatments (Vizoso et al., 2017). Exosomes are small (40–150 nm) membrane-enclosed particles derived from cellular endosomes that can transfer cargos including mRNAs, proteins, and other macromolecules between cells (Tkach and Théry, 2016). Notably, exosomes exhibit many of the same regenerative properties associated with stem cells, yet are not subject to many of the same limitations associated with stem cell-based therapies (Basu and Ludlow, 2016). Yao et al. found that the injection of BMSCs or BMSCs-derived exosome (BMSCs-exo) into the uterus in a rabbit model of uterine adhesions was sufficient to reduce endometrial fibrosis and to increase numbers of endometrial glands, with both of these approaches being similarly efficacious, underscoring the promise of exosome-based endometrial regeneration (Yao et al., 2019). While other studies have further confirmed the promising effects of exosomes as tools capable of attenuating endometrial fibrosis and promoting endometrial regeneration (Xiao et al., 2019; Lv et al., 2020; Saribas et al., 2020; Xin et al., 2020; Zhao et al., 2020), the benefits of these exosomes are relatively limited, highlighting an opportunity for the genetic modification-based enhancement of these therapeutic outcomes.

Cardiotrophin-1 (CTF1) is a cytokine in the interleukin (IL)-6 family that exhibits a range of cell type-dependent effects (López-Yoldi et al., 2015). While best studied in the context of cardiac function, CTF1 has recently been shown to exert protective activity in the renal, hepatic, and nervous systems (Stejskal and Ruzicka, 2008; López-Yoldi et al., 2015). For example, Yang et al. determined that CTF1 was able to enhance tissue regeneration in the context of liver cirrhosis by promoting cellular proliferation and angiogenesis (Yang et al., 2008), consistent with evidence that CTF1 overexpression using an adenoviral vector can improve liver function and survival associated with small-for-size grafts (Song et al., 2008). Such CTF1 expression has been shown to drive the proliferative, migratory, and angiogenic activity of endothelial cells *via* the ADMA/DDAH pathway (Zheng et al., 2015). Kobayashi et al. (2014) further employed an *in vivo* functional screening approach that identified CTF1 as an important promoter of successful embryo implantation. We therefore hypothesized that CTF1 overexpression would increase the proangiogenic activity of BMSCs-exo and the rates of successful pregnancy outcomes.

## Methods

### Animals

Female Sprague-Dawley (SD) rats (8-weeks-old; 200–250 g) were used for this study, and were housed in a climate-controlled facility with free access to food and water. All experiments were approved by the Institutional Animal Care and Use Committee of the Shanghai Jiao Tong University, and were consistent with the NIH Guide for the Care and Use of Laboratory Animals (NIH Publications No. 80-23).

### BMSC Culture

Bone marrow samples were isolated by flushing the femurs, tibiae, and humeri of C57BL/6 mice (female, 8 weeks old) with cold PBS. The bone marrow cells were passed through 70 um mesh filters (BD Biosciences, United States) and were then cultured in DMEM containing 10% fetal bovine serum (FBS, Gibco, MA, United States) and 1% streptomycin/penicillin (Sigma, MO, United States). BMSCs from passage 3 were used for downstream experiments.

### Analysis of BMSC Differentiation

A murine Mesenchymal Stem Cell Functional Identification Kit (Cyagen, SuZhou, China) was used to evaluate the ability of isolated BMSCs to undergo *in vitro* differentiation. Briefly, BMSCs were added to 6-well plates (10^5^ cells/well) in media containing adipogenic or osteogenic differentiation medium once 70–80% confluent. Supernatants were replaced every 2–3 days. On day 21, Alizarin Red staining and Oil Red O staining were used to assess osteoblastic and adipogenic differentiation, respectively (Liu et al., 2020). Cells were imaged *via* fluorescence microscopy (Olympus, Tokyo, Japan).

### Adenoviral Transduction

An adenoviral vector capable of promoting stable CTF1 overexpression was initially constructed. Briefly, the CTF1 full-length gene was cloned *via* RT-PCR, with the GFP-encoding pDC316-CMV construct being utilized as a shuttle vector. Following nuclease digestion, the sequence of the pDC316-CTF1 construct was confirmed. The adenovirus packaging plasmid (pBHGlox_E1.3Cre) was then co-transfected into HEK293 cells along with this construct, yielding the GFP-CTF1 adenoviral vector encoding CTF1, as confirmed *via* PCR. Following purification, amplification, and titration, this GFP-CTF1 adenovirus was used to infect BMSCs, after which CTF1 expression levels were confirmed *via* qPCR and Western blotting. An adenoviral vector encoding GFP served as a negative control construct. Infections were conducted for 24 or 48 h at multiplicity of infection (MOI) values of 250, 500, and 1,000 in order to optimize transduction efficiency.

### Exosome Isolation

Exosomes isolated from BMSCs or CTF1-BMSCs were respectively referred to as BMSCs-exo and C-BMSCs-exo. Briefly, once cells were 90% confluent, the overlying culture medium was discarded, and cells were rinsed once with PBS, after which media containing 10% exosome-depleted FBS and penicillin/streptomycin was added and cells were cultured for 48 h. Conditioned media was then collected and used to isolate the exosomes therein. Briefly, media was first centrifuged for 10 min at 400 × g to remove remaining cells, after which debris was removed by spinning supernatants for 20 min at 2,000 × g at 4°C. Samples were then centrifuged for 40 min at 15,000 × g, and supernatants were passed through a 0.22-μm filter (Millipore, MA, United States) prior to ultracentrifugation for 70 min at 120,000 × g. Exosomes were then washed with PBS and this ultracentrifugation step was repeated to isolate exosomes. Samples were maintained at or above 4°C at all times. A BCA Protein Assay Kit (Beyotime, Shanghai, China) was used based on provided directions to assess exosome protein concentrations, after which these particles were characterized *via* Western blotting, Nanoparticle Tracking Analysis (NTA), and transmission electron microscopy (TEM) (Wang et al., 2019).

### Animal Model Treatment

All rats were anesthetized by intraperitoneal injection of 2% sodium pentobarbital (0.3 ml/100 g), and the uterine horns were exposed *via* an abdominal incision. The proximal and distal portions of the uterus were then gently clamped with two vascular clamps, after which 95% ethanol was injected into the uterine cavity for 60 s. Vascular clamps were then released, gently squeezed to remove residual ethanol, and damaged sites were washed with PBS thrice to further remove remaining ethanol, as discussed in prior reports (Lin et al., 2021). HyStem-HP hydrogel (Catalog: GS315, Glycosan Biosystems, UT, United States) was utilized as a carrier for exosome delivery. This hydrogel was selected as it is composed of thiol-modified hyaluronan, HA, and thiol-modified heparin, and can undergo cross-linking *in situ* upon injection (Qin et al., 2016; Zhang et al., 2019). A total of 80 rats (*n* = 6–8 rats per group from three independent experiments) were assigned at random to four treatment groups (*n* = 20 for each group, 10 were sacrificed for HE, Masson and immunofluorescent evaluation and the other 10 for pregnancy test): 1) a sham-operated control group in which the uterine horn was injected with PBS (200 μl); 2) a model group in which the uterine horn was injected with 95% ethanol as in prior studies (Lin et al., 2021); 3) a model group that was treated with BMSCs-exo (100 μg/ml) in a hydrogel suspension *via* injection into the uterine horn (20 μg in 200 μl hydrogel); and 4) a model group that was treated with C-BMSCs-exo (100 μg/ml) in a hydrogel suspension that was injected into the uterine horn (20 μg in 200 μl hydrogel). For both exosome-treated groups, exosomal suspensions were administered 30 min after ethanol-mediated damage was induced. When three estrus cycles had passed after hydrogel injection, rats were euthanized and samples of uterine tissue were collected and frozen or sectioned for downstream analyses.

### Migration and Wound Healing Assays

For wound healing assays, human umbilical vein endothelial cells (HUVECs; ATCC) (4 × 10^4^/well) were added to the lower chamber of a well containing a Transwell assay insert (8-μm pores, Corning, United States), with 20 μg of BMSCs-exo or C-BMSCs-exo being added into the upper compartment. The monolayer was scratched with a sterile pipette tip, washed with PBS, and cells were then cultured for 24 h in high-glucose DMEM, with cells being imaged at 0, 12, and 24 h *via* inverted microscope (Olympus Microscopes, Tokyo, Japan). Wound closure in six randomly selected fields of view was then quantified using Photoshop (Adobe Systems Inc., CA, United States), with data being normalized to mean values in the control group.

For migration assays, HUVECs (4 × 10^4^) were instead added to the upper chamber of the Transwell insert, with BMSCs-exo or C-BMSCs-exo (20 μg) being added in the lower chamber. Following incubation for 18 h at 37°C, cells that had migrated to the lower chamber were fixed, stained with 0.5% crystal violet, and quantified *via* microscopy.

### Proliferation Assays

HUVEC proliferation was assessed *via* immunofluorescent Ki67 staining. Briefly, cells were fixed using 4% paraformaldehyde, permeabilized using 0.1% Triton X-100, and stained using anti-Ki67 (1:200; Abcam), followed by staining with an appropriate secondary antibody conjugated to AF488 (Abcam). Nuclear counterstaining was then performed, and Ki67-positive cells were quantified using the ImageJ program.

### Tube Formation Assays

To assesses the impact of BMSCs-exo or C-BMSCs-exo on endothelial tube formation, HUVECs (1 × 10^4^/well) were plated in 24-well plates on growth factor-reduced Matrigel (BD Biosciences, United States) for 16 h in the presence of appropriate exosomes, after which tubes were imaged *via* microscopy and randomly chosen pictures were analyzed with the Image J Angiogenesis Analyzer function.

### Fertility Analyses

Embryo implantation was measured as a correlate for endometrial receptivity. Briefly, rat estrus cycle progression was assessed *via* a vaginal smear approach. At 8 weeks post-surgery, female rats (10 rats, 20 uterine horns per treatment group) were mated with sexually mature male rats at a 1:1 ratio on the day of estrus, with rats being examined the following morning for the presence of a vaginal plug. On day 18 after the observation of a vaginal plug, rats were euthanized, and the size, number, and weight of fetuses therein were assessed, as were the locations of these fetuses.

### Histological and Immunofluorescent Staining

Endometrial tissue sections were fixed overnight in 4% paraformaldehyde, after which they were embedded in paraffin blocks and cut into 8-μm-thick tissue sections using previously described methods (Hashimoto et al., 2016). Sections were then stained with hematoxylin and eosin (H&E) or were subjected to Masson’s trichrome staining based upon provided protocols. Endometrial sections were additionally stained for CD31 and α-SMA as detailed previously (Qin et al., 2013), with DAPI as a nuclear counterstain.

### Western Blotting for Signaling Pathways

Total protein was isolated from the HUVECs co-cultured with 100 μg/ml BMSC-exos and 100 μg/ml CTF1-BMSC-exos for 48 h *in vitro*, and endometrial tissues of the BMSC-exo treatment group and the C-BMSC-exo treatment group *in vivo*, after which protein levels in the prepared lysates were measured. Protein was then separated *via* SDS-PAGE, transferred to PVDF membranes, and the resultant blots were probed overnight with antibodies specific for mTOR, p-mTOR, JAK, p-JK, PI3K, p-PI3K, STAT3, p-STAT3, AKT, p-AKT, or β-actin (1:500, Abcam) at 4°C. Relative protein band density was then assessed with an Odyssey infrared imaging system (LI-COR, NE, United States). The details of the quantification process of density values are specified in Image J software. Quantification of protein was normalized to β-actin.

### Flow Cytometry

Isolated BMSCs were characterized by staining them with antibodies specific for CD29 (Biolegend), CD34 (eBioscience), CD44 (Biolegend), CD45 (Biolegend), and CD90 (Biolegend) in a 100 μl volume of FACS buffer (Sigma, CA, United States). After two washes with FACS buffer, cells were fixed in Cytofix (BD Pharminigen, CA, United States) and analyzed with an ACEA NovoCyte flow cytometer. The FlowJo software was used for all data analyses.

### Statistical Analysis

Data are means ± SD and were analyzed *via* one-way ANOVAs with Tukey’s multiple comparison test using SPSS 22.0. *p* < 0.05 served as the significance threshold. In all figure legends, “*n*” represents the number of samples or rats utilized in the indicated experiments and technical and biological replicates are specified for each experiment.

## Results

### BMSC-Exo Isolation and Characterization

**FIGURE 1:**
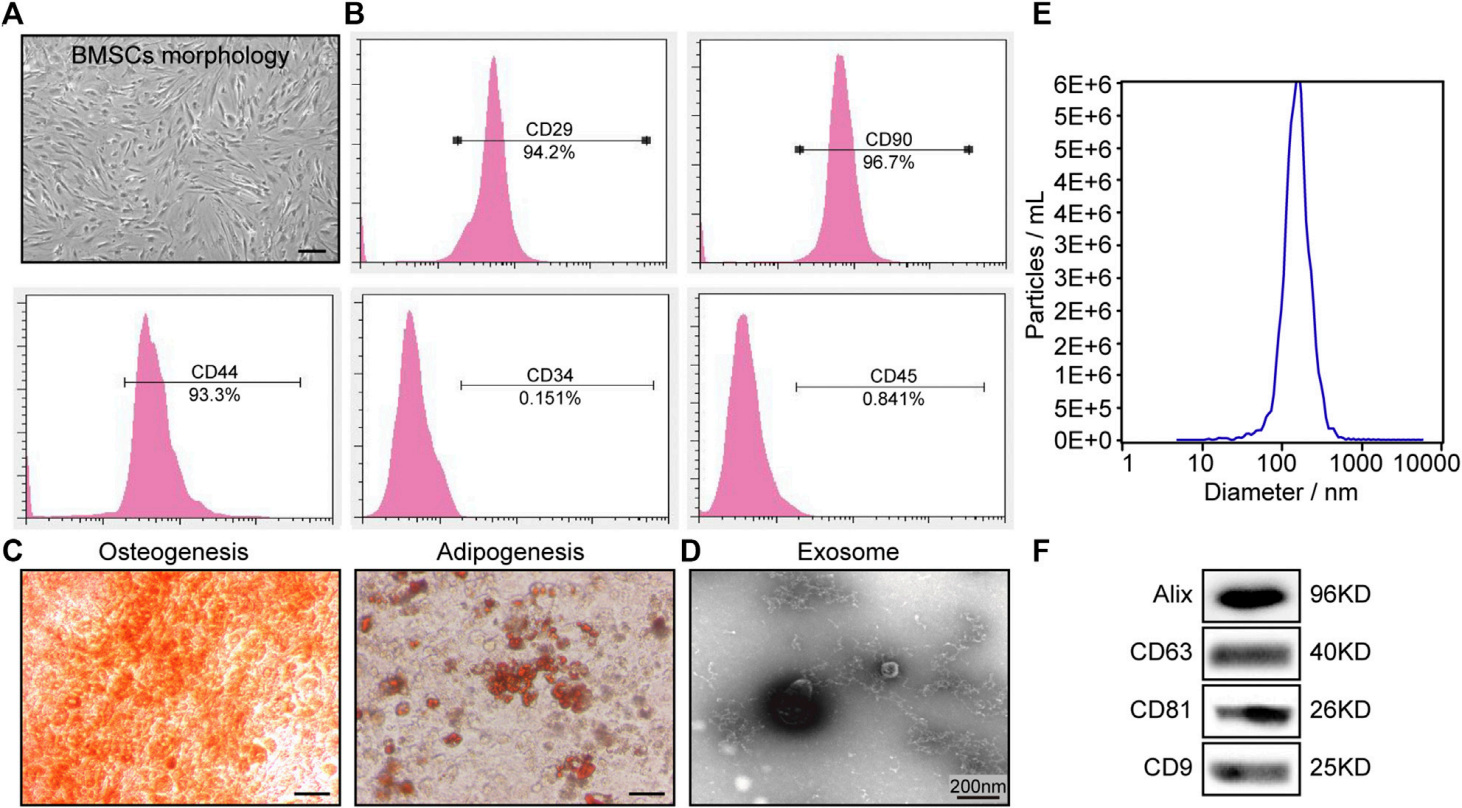
BMSC and BMSC-exo characterization. **(A)** Representative images of BMSCs following culture for three passages. **(B)** Assessment of BMSC staining for CD29, CD34, CD44, CD45, and CD90 *via* flow cytometry. **(C)** Representative images of BMSCs following osteogenic or adipogenic differentiation. Scale bar = 200 µm. **(D)** TEM images of BMSC-exos. Scale bar = 200 nm. **(E)** BMSC-exo size distribution plot. **(F)** Western blotting was used to measure Alix, CD63, CD81, and CD9 levels in BMSC-exo preparations.

First, we isolated BMSCs from C57BL/6 mice and culturing these cells for three passages, after which cells were used for downstream experiments (Figure 1A). Flow cytometry confirmed that these cells were negative for hematopoietic markers (CD34) and were positive for BMSC markers antigens (CD29, CD90, CD44, and CD45) (Figure 1B). Consistent with their identity as multipotent progenitor cells, these BMSCs were able to differentiate into osteoblast- and adipocyte-like cells when treated with appropriate differentiation media, as confirmed *via* Alizarin Red staining and Oil Red O staining, respectively (Figure 1C). BMSCs-derived exosome were then collected *via* differential centrifugation from BMSC-condition media. TEM analyses confirmed these BMSCs-exo to exhibit rounded or cup-shaped morphologic characteristics consistent with those of exosomes (Figure 1D), while TRPS measurements indicated these exosomes to be 60–120 nm in size (Figure 1E). Western blotting also revealed these particles to be positive for exosomal surface markers including Alix, CD9, CD63, and CD81 (Figure 1F). Together, these data confirmed that we had successfully enriched mouse BMSCs-derived exosome.

### Genetically Modified BMSC Preparation

**FIGURE 2:**
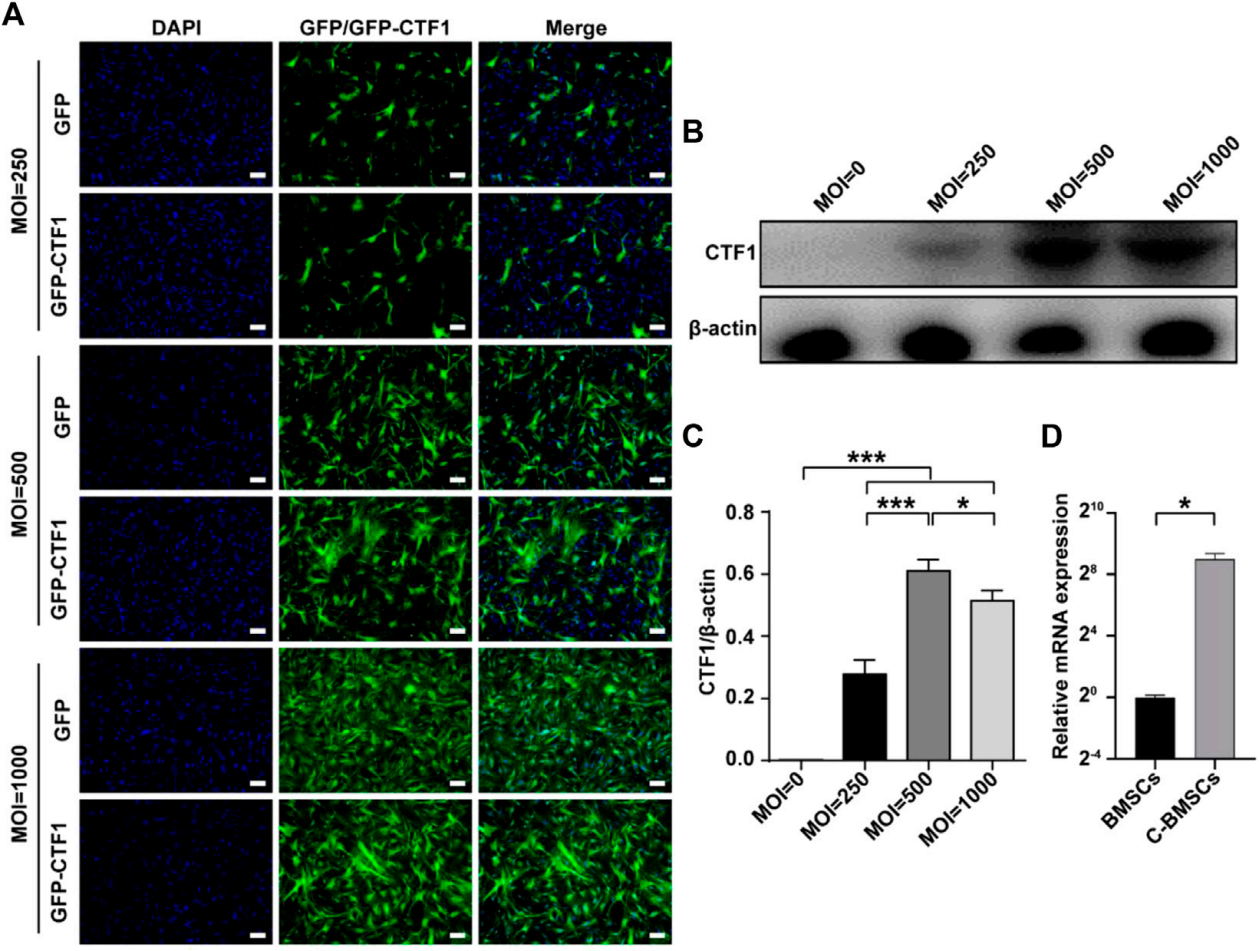
Stable adenoviral overexpression of CTF1 in BMSCs. **(A)** Transduction ratios for cells overexpressing CTF1 or GFP (as a control). **(B–D)** Western blotting (*n* = 3, independent biological replicates) and qPCR (*n* = 3, technical replicates) were used to confirm the increased expression of CTF1 in C-BMSCs relative to control BMSCs (*p* < 0.05). Scale bar = 100 μm. *p < 0.05, **p < 0.01, ***p < 0.001.

An adenoviral vector was used to stably overexpress CTF1-GFP in murine BMSCs as detailed in the *Materials and Methods* section, with GFP overexpression serving as a negative control. A range of adenoviral transduction intervals and MOI values were tested to maximize the efficiency of this overexpression approach (Figure 2).

An adenoviral vector was used to transduce CTF1 into BMCSc at an MOI of 500, with maximal transduction efficiency being evident after 48 h (Figure 2). The transduction ratio did not vary markedly between CTF1 and GFP in BMSC cells (Figure 2A). Western blotting and PCR confirmed that these CTF1-transduced BCSCs exhibited significant increases in CTF1 expression, whereas it was almost undetectable in the control BMSC group (*p* < −0.05) (Figures 2B–D).

### C-BMSC-Exo Treatment Alters the Proliferative, Migratory, and Angiogenic Activity of HUVECs *In Vitro*

**FIGURE 3:**
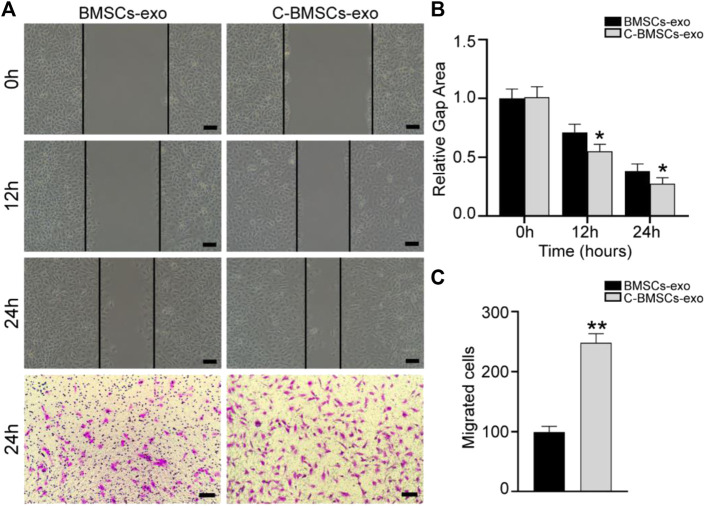
C-BMSCs-exo treatment alters HUVEC migration. **(A)** Representative wound healing and Transwell assay results following culture for 24 h. **(B)** Wound healing assays revealed significantly enhanced wound healing in the C-BMSC-exo group relative to the BMSC-exo group (*n* = 3). **(C)** Transwell assays revealed significantly more migrator cells in the C-BMSC-exo group relative to the BMSC-exo group (*n* = 3). *p < 0.05, Scale bar = 100 mm.

Angiogenesis is an essential process whereby stem cell-derived exosomes can drive endometrial regeneration. As such, we next evaluated the effects of C-BMSC-exo treatment on the proliferation, migration, and tube formation activity of HUVECs *in vitro.* In a wound healing assay, C-BMSC-exo treatment was associated with significant improvements in wound closure at 12 and 24 h time points relative to BMSC-exo treatment (*p* < 0.05; Figures 3A,B). In migration assays, C-BMSC-exo treatment was associated with significant increases in cellular migration relative to BMSC-exo treatment (99 ± 4 vs. 247 ± 4 cells/field; *p* < 0.05) (Figures 3A,C). These data thus suggested that CTF1 overexpression was linked to enhanced migratory activity upon BMSC-exo treatment.

**FIGURE 4:**
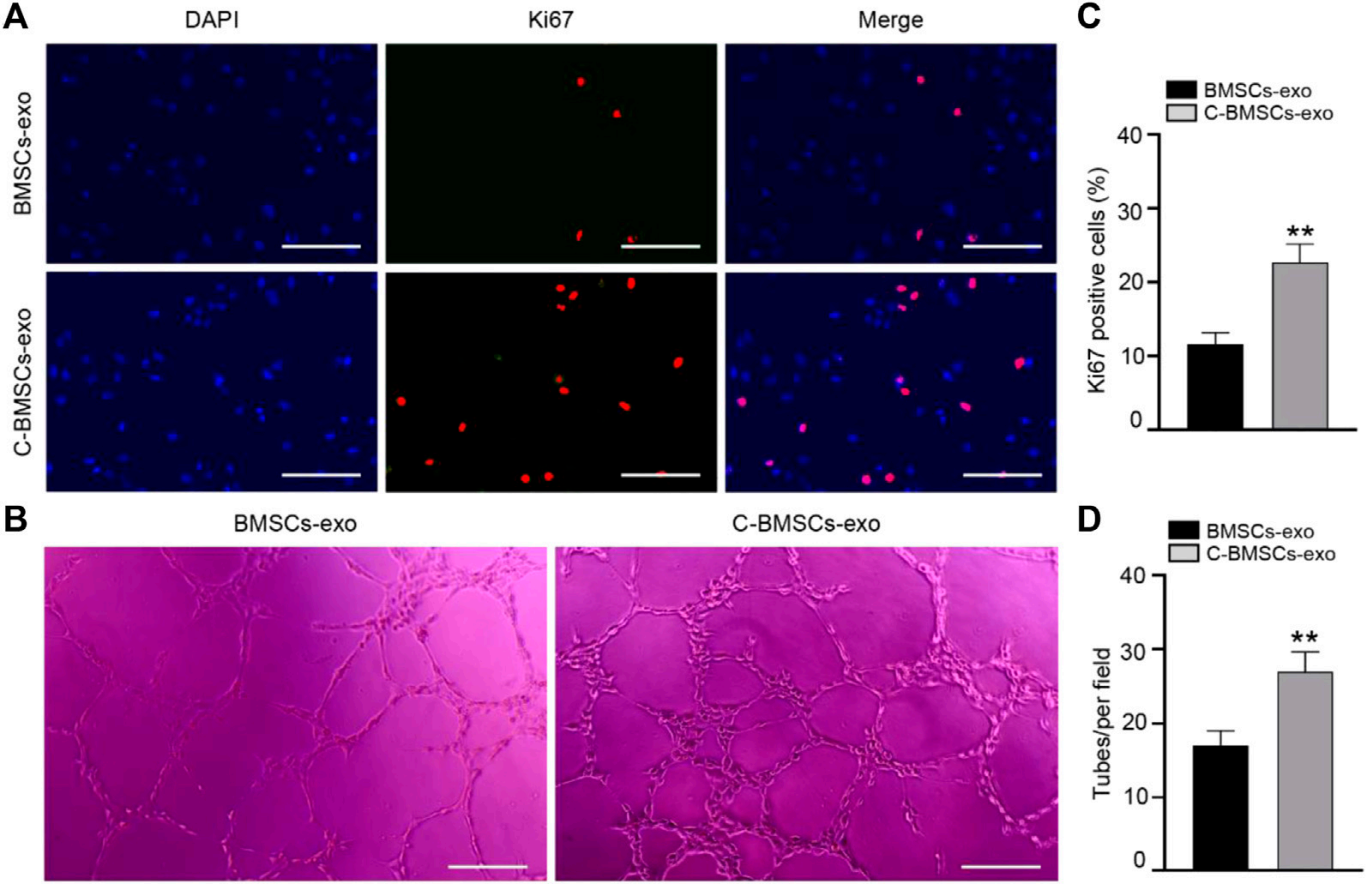
C-BMSC-exo treatment alters HUVEC proliferation and angiogenic activity. **(A)** Ki67 staining was used to assess HUVEC proliferation in the indicated groups (Scale bar: 50 μm). **(B)** Representative images of HUVEC tube formation following culture on Matrigel for 12 h in the presence of C-BMSC-exo or BMSC-exo treatments. **(C)** Numbers of Ki67-positive cells were quantified (*n* = 3, biological replicates). **(D)** Numbers of tubes per field of view were quantified, indicating that C-BMSC-exo treatment enhanced tube formation (Scale bar:200 μm) (*n* = 3, biological replicates). Samples were analyzed in triplicate. *p < 0.05, **p < 0.01.

In proliferation assays, Ki67 staining was performed following a 3-day treatment, revealing that the majority of cells in both the BMSC-exo and C-BMSC-exo groups were Ki67-positive (Figure 4A), with C-BMSC-exo treatment being associated with greater positivity relative to BMSC-exo treatment (Figure 4C). These results thus indicated that C-BMSC-exo treatment was sufficient to promote sustained *in vitro* endothelial cell proliferation.

Tube formation assays were next conducted to evaluate the angiogenic potential of treatment with these different exosomal preparations. In line with the above data, we found that C-BMSC-exo treatment was associated with significantly enhanced tube formation relative to BMSC-exo treatment (Figures 4B,D). Overall, these findings suggested that CTF1 overexpression led to enhanced proangiogenic activity following BMSC-exo treatment.

### C-BMSC-Exo Treatment Enhances Endometrial Regeneration and Prevents Fibrosis *In Vivo*

**FIGURE 5:**
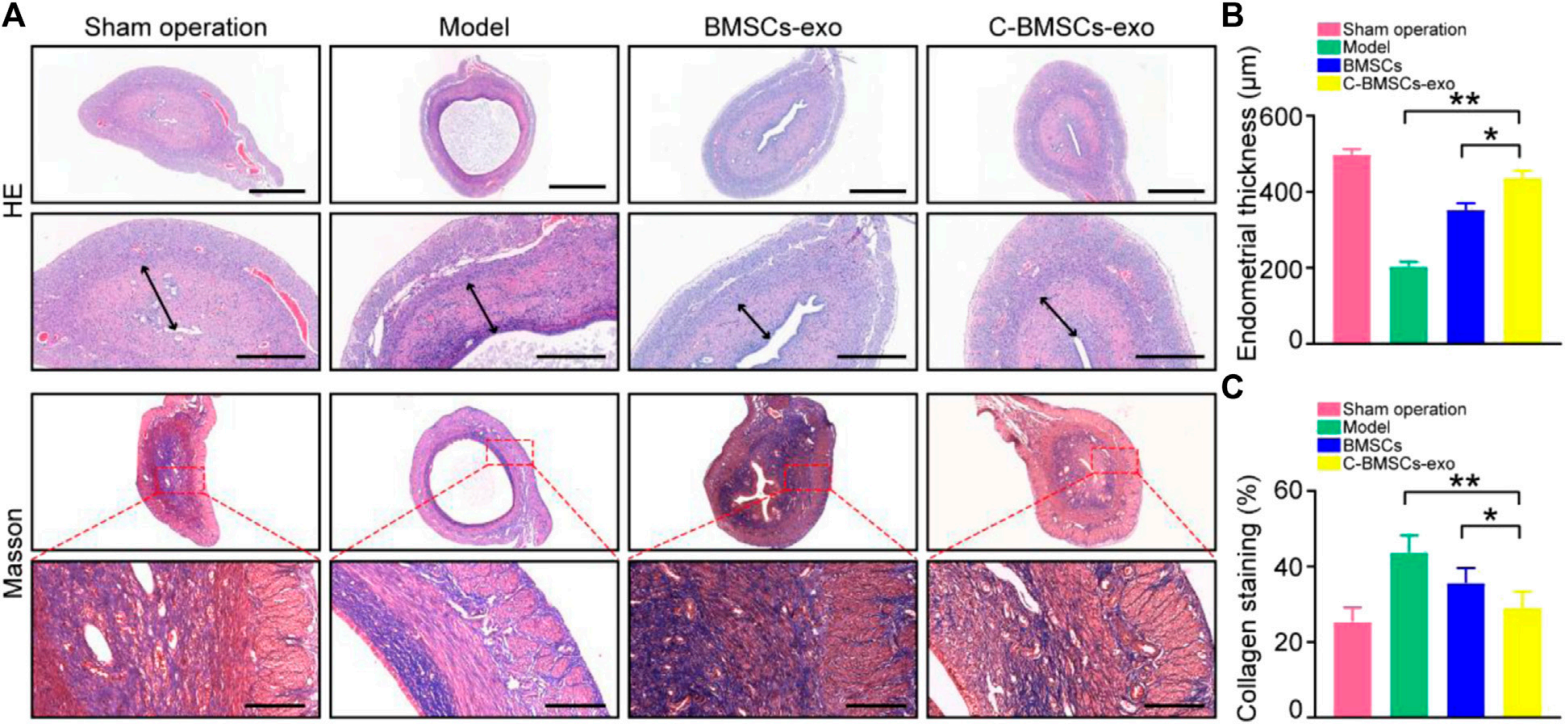
BMSC-exo or C-BMSC-exo treatment promotes endometrial regeneration and reduces endometrial fibrosis. **(A)** H&E staining was used to assess myometrial regeneration (Scale bars: 1,000 µm, 200 µm), while endometrial fibrosis was assessed *via* Mason’s trichrome staining, with scar formation being shown in blue (Scale bar: 100 μm). **(B)** Endometrial thickness in the indicated groups was quantified (*n* = 3 mice per group from three independent experiments). **(C)** Collagen protein levels in the indicated groups were quantified (*n* = 3 mice per group from three independent experiments). Data are means ± Standard deviation, and samples were assessed in triplicate. *p < 0.05, **p < 0.01.

We next employed an intrauterine anhydrous ethanol injection approach to model endometrial damage *in vivo* in rats. Endometrial damage was assessed in these animals *via* a series of histological approaches after three estrus cycles had passed following damage. Endometrial damage and thinning were evident in model group rats such that in some cases the endometrium and myometrium could not be distinguished from one another. Significant endometrial regeneration was evidence in rats which had been injected with C-BMSC-exo preparations, as evidenced by increased angiogenesis and areas of well-distributed cells (Figure 5A). Such treatment was also associated with appropriately structured stromal tissues, epithelial layers, and secretory glands. Rats in the sham-operated control, model, BMSC-exo group and C-BMSC-exo group exhibited endometrial tissues with a thickness of 490, 200, 350, and 425 µm, respectively, underscoring the potent regenerative effects of C-BMSCs-exo treatment (Figure 5B).

Masson’s trichrome staining was next conducted to assess collagen deposition within uterine tissue sections from these treated rats, revealing significant reductions in endometrial fibrosis and enhanced muscle fiber growth following BMSCs-exo treatment (Figure 5C). Importantly, C-BMSCs-exo treatment was associated with even more robust anti-fibrotic activity (Figure 5C), suggesting that C-BMSCs-exo can effectively prevent tissue damage and fibrosis, thus promoting efficient vascular and glandular proliferation following the induction of endometrial damage.

### C-BMSC-Exo Treatment Promotes Angiogenesis and Endometrial Regeneration *In Vivo*

**FIGURE 6:**
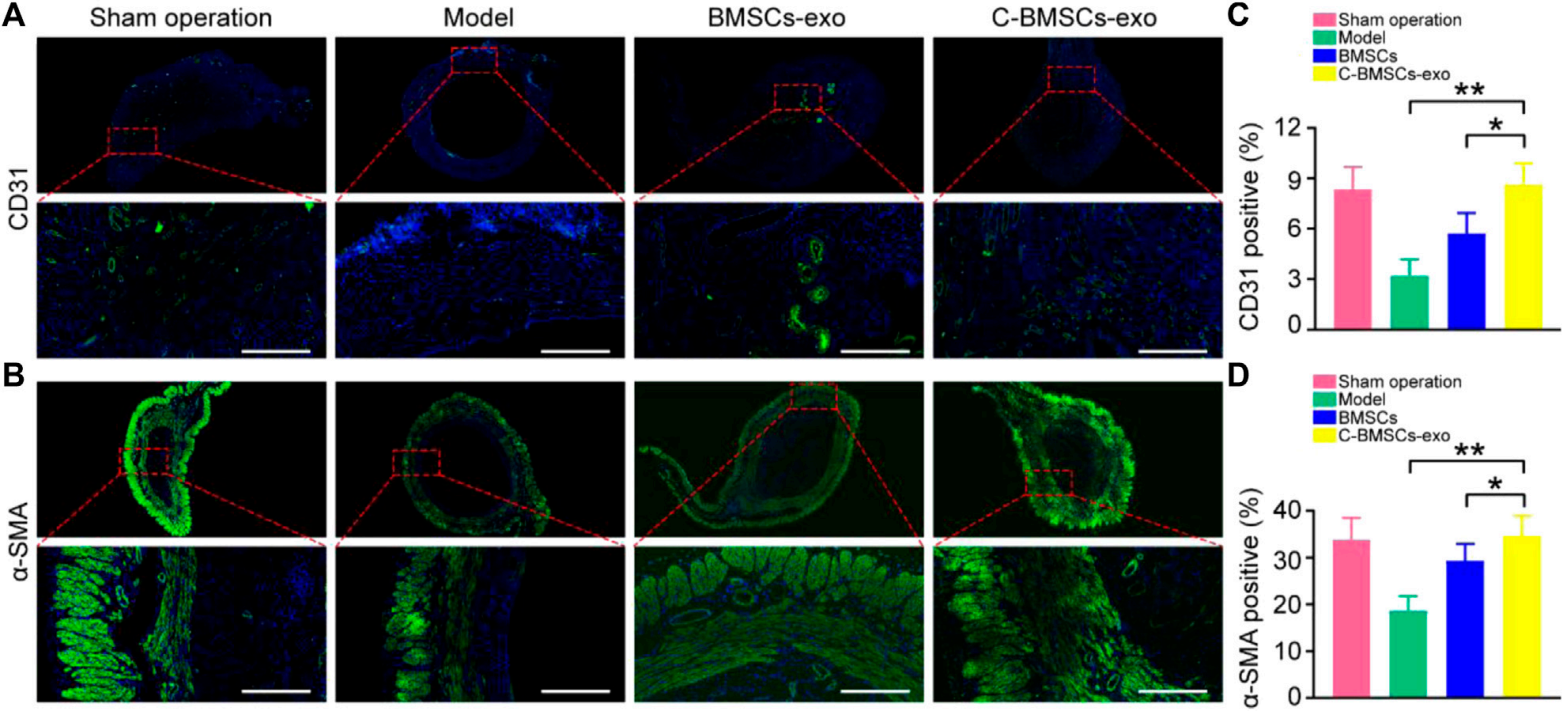
BMSCs-exo or C-BMSCs-exo administration induces endometrial neovascularization and myometrial regeneration. **(A)** CD31-positive endothelial cells were detected *via* immunofluorescent staining in the indicated treatment groups (Scale bar: 100 µm). **(B)** Smooth muscle was assessed *via* α-SMA staining to assess myometrial regeneration (Scale bar: 100 µm). **(C,D)** CD31 and α-SMA expression were quantified (*n* = 3 mice per group from three independent experiments). Data are means ± Standard deviation. *p < 0.05, **p < 0.01.

Endometrial regeneration is dependent on angiogenesis, which facilitates oxygen and nutrient delivery to damaged tissues (Gurtner et al., 2008). As such, we next explored the effects of C-BMSCs-exo treatment on *in vivo* neovascularization (Figure 6). We therefore stained endometrial tissues with the anti-CD31 to detect endothelial cells, revealing that there were significantly higher levels of CD31 staining in the BMSCs-exo and C-BMSCs-exo treatment groups as compared to the model group (Figure 6A), C-BMSCs-exo treatment being associated with the highest levels of staining. Immunofluorescent staining results further confirmed that C-BMSCs-exo treatment resulted in significantly increased *in vivo* neovascularization (Figure 6C).

Anti-α-SMA was next used to stain tissues in order to assess myometrial regeneration (Figure 6B). Animals in the control and BMSCs-exo groups exhibited a thin, continuous layer of circular muscle fibers, whereas this was not the case in the model groups. Rats in the C-BMSCs-exo group exhibited a significant increase in the number of muscle bundles as compared to the BMSCs-exo group (Figure 6B). The α-SMA staining area in the C-BMSC-exo group was significantly higher than that in the BMSCs-exo group (Figure 6D). Together, these data indicated that BMSCs-exo treatment promoted myometrial regeneration.

### C-BMSCs-Exo Treatment Leads to Improved Fertility Outcomes

**FIGURE 7:**
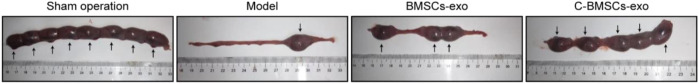
C-BMSC-exo treatment enhances fertility restoration. Endometrial regeneration was assessed based upon the efficiency of embryo implantation, with pregnancies being assessed in the indicated groups at 8 weeks post-surgery.

To further evaluate the functional effects of C-BMSC-exo treatment on endometrial regeneration, we next measured implantation rates in different treatment groups by counting embryos in rats on gestational day 18 (Figure 7; Supplementary Table S1). Sham-operated control rats exhibited the highest implantation rates, but the rates in the C-BMSC-exo treatment group were significantly higher than those in the untreated and BMSC-exo groups (Figure 7), suggesting the ability of C-BMSC-exo treatment to restore fertility by promoting functional endometrial regeneration.

### Evaluation of the Signaling Pathways Associated With C-BMSCs and C-BMSC-Exos

**FIGURE 8:**
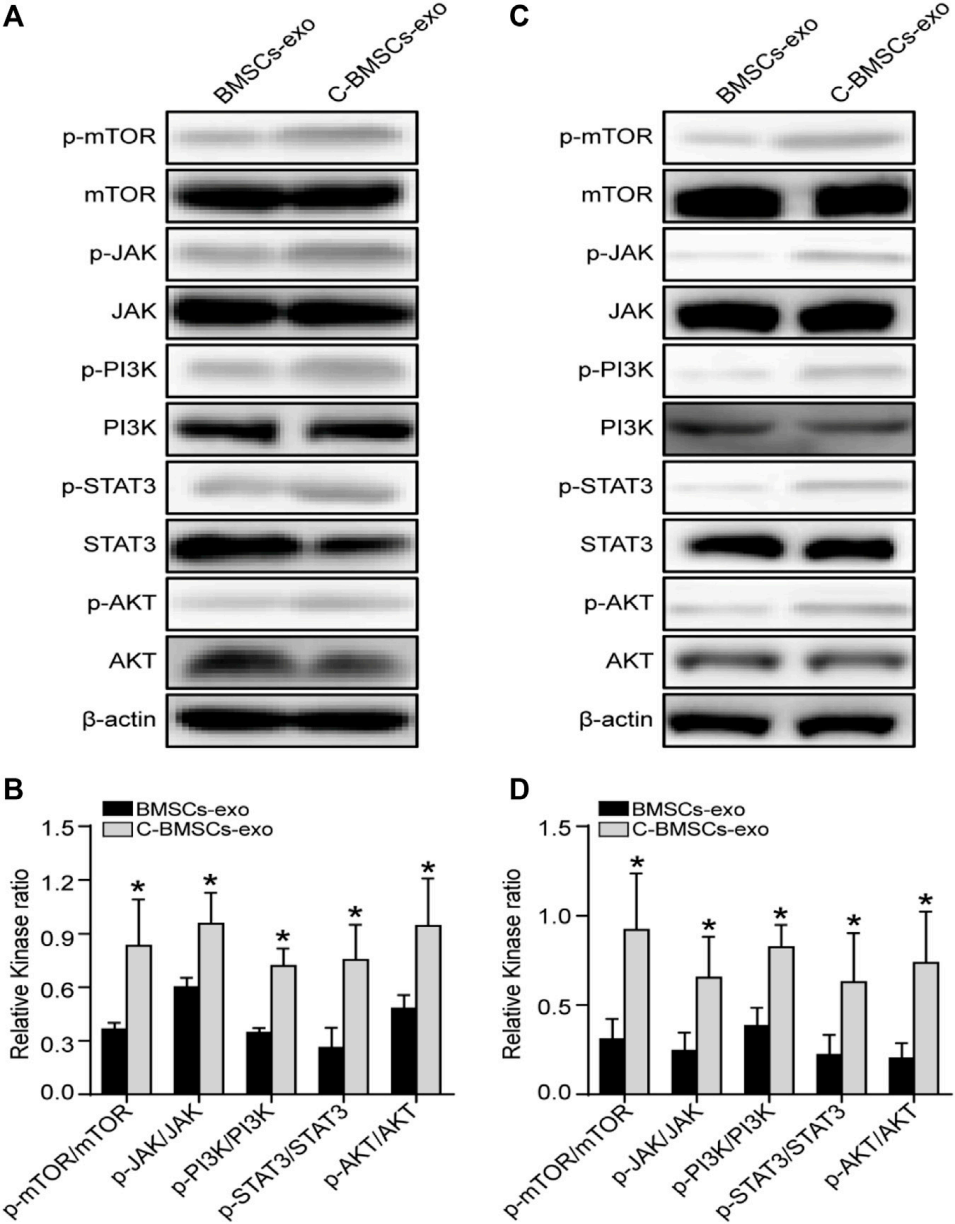
Assessment of the *in vitro* and *in vivo* effects of CTF1 in the context of BMSC-exo treatment. **(A)** Western blotting was used to assess JAK, p-JAK, PI3K, p-PI3K, AKT, p-AKT, mTOR, p-mTOR, STAT3, and p-STAT3 levels *in vitro* in the context of CTF1 expression in BMSCs-exo. **(B)** Statistical analyses demonstrating significant increases in JAK/PI3K/mTOR/STAT3 phosphorylation in C-BMSC-exo treatment conditions relative to BMSC-exo treatment (*n* = 3). **(C)** Western blotting was used to assess JAK, p-JAK, PI3K, p-PI3K, AKT, p-AKT, mTOR, p-mTOR, STAT3, and p-STAT3 levels *in vivo* in the context of CTF1 expression in BMSC-exo treatment. **(D)** Statistical analyses exhibiting significant increases in JAK/PI3K/mTOR/STAT3 pathway activation in the C-BMSCs-exo group relative to the BMSC-exo group (*n* = 3). *p < 0.05.

Lastly, we performed a series of Western blotting analyses examining the signaling pathways whereby CTF1 may mediate beneficial fertility outcomes. We found that C-BMSCs-exo groups exhibited significantly higher levels of JAK/PI3K/mTOR/STAT3 phosphorylation relative to BMSCs-exo control groups (Figures 8A,C), with these increases being confirmed *via* statistical analyses (Figures 8B,D). And no change in AKT expression was observed in the C-BMSCs-exo groups relative to the corresponding BMSC-exo controls. We speculated that STAT3 was a key signaling mediator in this context. Indeed, p-STAT3 has previously been reported as a regulator of VEGFR2 activity (Chen et al., 2008). CTF1 can promote angiogenesis and myocardial cell survival through the induction of VEGF-dependent myotubule formation (Freed et al., 2005). BMSCs-exo treatment with CTF1 can also result in rapid STAT3 phosphorylation, leading to increased cellular adherence (Bortolotti et al., 2017). There is also prior evidence in ICR (Institute of Cancer Research) and B6 (C57BL/6) mice that CTF1 can promote STAT3 phosphorylation in the uterine luminal epithelium, leading to higher implantation rates (Kobayashi et al., 2014). These data highlight the importance of CTF1 as a promoter of angiogenesis and persistence following BMSCs-exo treatment *in vitro* and *in vivo*, thereby leading to improved endometrial regeneration and increased endometrial receptivity.

## Discussion

Paracrine signaling is a key mechanism whereby stem cells promote endometrial repair *via* the release of key bioactive molecules (Yao et al., 2019; Lv et al., 2020). Exosomes and other extracellular vesicles derived from stem cells can drive endometrial regeneration and angiogenesis in a female mouse model system (Xiao et al., 2019; Saribas et al., 2020). We have previously shown that ADSC-derived exosomes were able to promote endometrial regeneration, fertility restoration, and increased rates of live birth (Lin et al., 2021). Herein, we developed an efficient approach to generating C-BMSCs-derived exosome using BMSCs overexpressing CTF1. We found that these C-BMSCs-exo were able to promote enhanced endometrial regeneration relative to BMSCs-exo and model control groups. C-BMSCs-exo treatment also enhanced endothelial cell proliferation, migration, and tube formation *in vitro* along with angiogenesis *in vivo.* Our analyses suggest that such angiogenic activity may be tied to the enhanced activation of the JAK/PI3K/mTOR/STAT3 signaling pathway.

Angiogenesis is a process wherein the basement membrane of existing blood vessels are degraded and endothelial cells are then able to migrate into this region to generate a branching vessel, with two of these vessel sprouts then joining together to yield a functional capillary loop, followed by subsequent vessel maturation (Abulafia and Sherer, 1999). Angiogenesis is a key mediator of endometrial regeneration, facilitating appropriate nutrient and oxygen delivery to healing tissues (Gurtner et al., 2008).

Wang et al. (2019) suggested that exosome from adipose-derived mesenchymal stem cells can activate HUVEC angiogenic responses *in vitro,* inducing the proliferation and migration of these cells while further promoting *in vivo* angiogenesis and wound healing. Hu et al. (2018) determined that human umbilical cord blood-derived exosomes were able to promote angiogenesis and fibroblast functionality and to thereby enhance cutaneous wound healing in a manner dependent upon miR-21-3p. Liu et al. (2019) confirmed that BMSC-derived factors were able to promote angiogenesis and epithelial/endothelial cell proliferation in a rat model of Asherman’s syndrome. We have previously shown that ADSCs-derived exosome can promote *in vitro* and *in vivo* angiogenesis (Lin et al., 2021). Recent work suggests that genetic modification can significantly enhance the regenerative and proangiogenic activity of MSC-derived exosomes. Ma et al. (2017), for example, determined that MSCs-derived exosome that had been modified to express AKT at the mRNA level were better able to promote angiogenesis and better therapeutic outcomes in the context of myocardial infarction relative to unmodified MSCs-exo. Sun et al. (2020) determined that HIF-1α-overexpressing MSCs-derived exosome exhibited increased cardioprotective activity in infarcted tissue relative to unmodified MSCs-exo exhibited overall better therapeutic effects than MSCs-exosome. Herein, we similarly found that C-BMSCs-exo treatment was associated with enhanced angiogenesis *in vitro* and *in vivo* and better endometrial repair outcomes relative to BMSCs-exo treatment.

CTF1 has been identified as an important regulator of cardiac, renal, hepatic, and nervous system functionality that has also recently been detected in the murine endometrium during the early stages of pregnancy (Kobayashi et al., 2014). At a functional level, this cytokine is known to promote endothelial cell and neuronal survival, proliferation, migration, and differentiation, and it can also promote MSC persistence following injection into a particular tissue site (Zheng et al., 2015; Bortolotti et al., 2017). Yang et al. (2008) determined that CTF1 treatment was linked to transient increases in microvessel density and VEGF production, suggesting that these two cytokines influence one another to drive angiogenic processes. Indeed, there is evidence that CTF1 can promote angiogenesis in large part by driving VEGF/VEGFR pathway activation in diseased hepatocytes. CTF1 treatment has also been shown to improve embryo implantation in ICR and B6 mice by activating transcriptional activity within the uterine luminal epithelium (Kobayashi et al., 2014). In the current study, we extended these prior results by demonstrating that CTF1 improved angiogenesis and endometrial receptivity in the context of BMSC-exo treatment.

In one prior report, hypoxia-induced HIF-1α and CTF1 were shown to influence cellular proliferation, survival, and glucose uptake *via* the PI3K/AKT promote pathway (Alvarez-Tejado et al., 2001). In other reports, mTOR signaling has been identified as a critical regulator of angiogenesis and endothelial repair (Nakao et al., 2007), mediating the production of a range of critical growth factors (Meng et al., 2019). STAT3, in turn, functions as a downstream component of the mTOR signaling pathway (Meng et al., 2019), with STAT3 signaling being linked to angiogenic activity (Nakamura et al., 2015). Our data support an important role for this JAK/PI3K/mTOR/STAT3 signaling pathway in the context of endometrial receptivity and regeneration through the promotion of cellular proliferation, migration, and angiogenesis. However, our analyses on this topic were relatively cursory, and further mechanistic research will be vital to fully elucidate the pathways whereby C-BMSCs-exo treatment achieves therapeutic efficacy. Despite these limitations, our study provides clear evidence that CTF1 can enhance angiogenesis, suppress tissue fibrosis, and enhance endometrial receptivity following BMSCs-exo treatment, highlighting a viable approach to treating damaged endometrial tissues.

## Conclusion

In summary, these data indicate that exosomes isolated from BMSCs that were genetically engineered to overexpress CTF1 can mediate more efficient repair of injured endometrial tissues by promoting enhanced angiogenesis and consequent improvements in endometrial receptivity. The mechanistic basis for the effects of these CTF1-modified BMSCs-exo was linked to JAK/PI3K/mTOR/STAT3 signaling pathway activation. Together, these results highlight a promising approach to enhancing endometrial regeneration and thereby improving fertility outcomes.

## Data Availability

The original contributions presented in the study are included in the article/Supplementary Material, further inquiries can be directed to the corresponding authors.

## Supplementary Material

Supplementary Table S1 Reproductive outcomes over 8 weeks following different treatments.

## Abbreviations

BMSCs, Bone marrow-derived stem cells; CTF1, Cardiotrophin-1; Exo, exosome; G-CSF, granulocyte colony-stimulating factor (G-CSF); VEGF, Vascular endothelial growth factor; GnRH, Gonadotropin-releasing hormone; HRT, Hormone replacement therapy; DMEM, Dulbecco’s modified Eagle’s medium; PBS, Phosphate-buffered saline; FBS, Fetal bovine serum; GFP, Green fluorescent protein; HEK293, Human embryonic kidney cell line 293; MOI, Multiplicity of infection; TEM, transmission electron microscope; C-BMSCs, Cardiotrophin-1-transfected BMSCs; C-BMSCs-exo, Cardiotrophin-1-transfected BMSCs exosome; HUVECs, Human umbilical vein endothelial cells; FITC, Fluorescein isothiocyanate.
