# Supplementary figures and images for: Exosomes Derived From CTF1-Modified Bone Marrow Stem Cells Promote Endometrial Regeneration and Restore Fertility
Source: Front Bioeng Biotechnol. 2022 Apr 13;10:868734. doi: 10.3389/fbioe.2022.868734 (PMC9043110; doi:10.3389/fbioe.2022.868734)

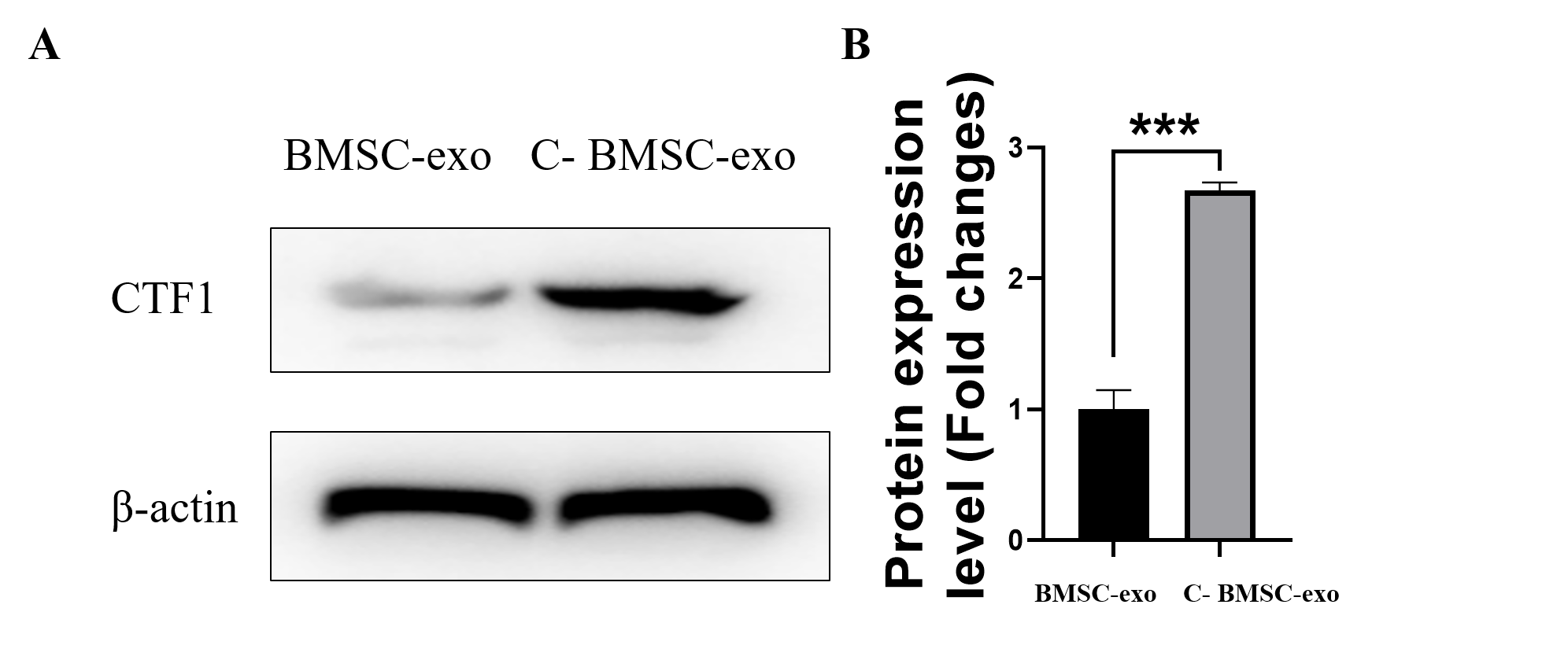

Supplement: Supplementary file 1 [file Image1.TIF]
